# Clofazimine induced pigmentation in leprosy patches

**DOI:** 10.11604/pamj.2022.42.14.34956

**Published:** 2022-05-09

**Authors:** Samiksha Deepak Chavhan, Sugat Jawade

**Affiliations:** 1 Department of Dermatology Venereology and Leprosy, Jawaharlal Nehru Medical College, Sawangi (Meghe), Wardha, Maharashtra, India

**Keywords:** Lipofuscin, hyperpigmentation, macrophage

## Image in medicine

Leprosy is a neurocutaneous infectious disease with a highest prevalence in African region in the world. With the introduction of newer monitoring strategies of identifying the cases and multidrug therapy the overall global burden of leprosy has reduced. However, early detection and treatment is still important in leprosy due to the rapidly developing nerve damage by the bacteria and the associated disability. Here, presenting a case of a 65-year-old male patient of leprosy in borderline pole, with complaint of hyperpigmentation over back, chest and arm in a ring like pattern from past 4 months. Patient has been on WHO multibacillary multidrug regimen (Rifampicin=600mg once a month, Clofazimine=100mg once a month + 50mg daily dose, Dapsone=100mg daily). On starting the treatment patient started noticing resolution in thickness of the lesion along with appearance of reddish-brown color of the lesion. On cutaneous examination, multiple geographic and annular patches with central clearing and shiny coppery reddish brown pigmentation in the peripheral margin of the lesion is seen. This case exemplifies the lesional hyperpigmentation, secondary to accumulation of ceroid lipofuscin pigment and Clofazimine inside the macrophage phagolysosome. Also, only marginal pigmentation with central clearing attributes to the punched-out lesion of the borderline lepromatous leprosy primarily which on histopathology typically shows presence of macrophage granuloma. Clofazimine induced pigmentation is reversible but takes months to years to clear after stopping the drug. As a dermatologist with leprosy moving towards elimination monitoring and managing the adverse effect of treatment and course of the disease is important.

**Figure 1:**
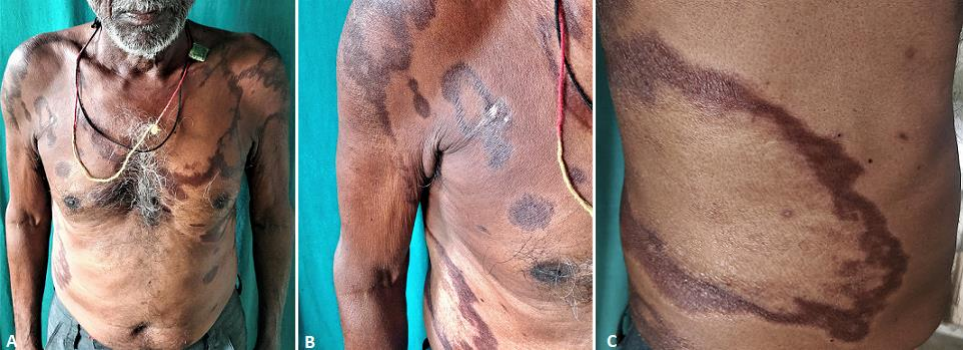
A) pigmentation patches seen over upper chest, arms and few over abdomen; B) annular pigmented patches with central clearing over right shoulder; C) large well demarcated patch with area of central clearing and peripheral pigmentation over back

